# Simultaneous use of solution NMR and X-ray data in *REFMAC*5 for joint refinement/detection of structural differences

**DOI:** 10.1107/S1399004713034160

**Published:** 2014-03-19

**Authors:** Mauro Rinaldelli, Enrico Ravera, Vito Calderone, Giacomo Parigi, Garib N. Murshudov, Claudio Luchinat

**Affiliations:** a Center for Magnetic Resonance (CERM), University of Florence, Via L. Sacconi 6, 50019 Sesto Fiorentino (FI), Italy; b Department of Chemistry ‘Ugo Schiff’, University of Florence, Via della Lastruccia 3, 50019 Sesto Fiorentino (FI), Italy; c MRC Laboratory of Molecular Biology, Francis Crick Avenue, Cambridge Biomedical Campus, Cambridge CB2 0QH, England

**Keywords:** structure refinement, PCS, RDC, X-ray, *REFMAC*

## Abstract

The program *REFMAC*5 from *CCP*4 was modified to allow the simultaneous use of X-ray crystallographic data and paramagnetic NMR data (pseudocontact shifts and self-orientation residual dipolar couplings) and/or diamagnetic residual dipolar couplings. Incorporation of these long-range NMR restraints in *REFMAC*5 can reveal differences between solid-state and solution conformations of molecules or, in their absence, can be used together with X-ray crystallographic data for structural refinement. Since NMR and X-ray data are complementary, when a single structure is consistent with both sets of data and still maintains reasonably ‘ideal’ geometries, the reliability of the derived atomic model is expected to increase. The program was tested on five different proteins: the catalytic domain of matrix metalloproteinase 1, GB3, ubiquitin, free calmodulin and calmodulin complexed with a peptide. In some cases the joint refinement produced a single model consistent with both sets of observations, while in other cases it indicated, outside the experimental uncertainty, the presence of different protein conformations in solution and in the solid state.

## Abstract 2

Paramagnetic NMR data (pseudocontact shifts and self-orientation residual dipolar couplings) and diamagnetic residual dipolar couplings can now be used in the program *REFMAC*5 from CCP4 as structural restraints together with X-ray crystallographic data. These NMR restraints can reveal differences between solid state and solution conformations of molecules or, in their absence, can be used together with X-ray crystallographic data for structural refinement.

## 1. Introduction   

Long-range paramagnetic NMR data such as pseudo-contact shifts (PCSs; Horrocks & Hall, 1971 ▶; Barry *et al.*, 1971 ▶; La Mar *et al.*, 1973 ▶) and/or self-orientation residual dipolar couplings (RDCs; Tolman *et al.*, 1995 ▶; Bothner-By, 1996 ▶) arising from a paramagnetic metal coordinated to the protein have been shown to be valuable restraints to help in solving protein structures in the solution state (Gochin & Roder, 1995 ▶; Banci *et al.*, 1996 ▶, 1998 ▶) and since then have been thoroughly used (Bertini *et al.*, 2001 ▶; Gaponenko *et al.*, 2004 ▶; Díaz-Moreno *et al.*, 2005 ▶; Pintacuda *et al.*, 2006 ▶; Jensen *et al.*, 2006 ▶; Schmitz *et al.*, 2012 ▶). PCSs measured for proteins in the solid state have also proved very useful to obtain both the molecular structure (Balayssac *et al.*, 2008 ▶; Bertini *et al.*, 2011 ▶), when used together with other solid-state data, and the relative arrangement of the molecules within the crystal (Luchinat *et al.*, 2012 ▶). Both PCSs and self-orientation RDCs can also be obtained by purposely attaching a paramagnetic tag to the protein (Wöhnert *et al.*, 2003 ▶; Rodriguez-Castañeda *et al.*, 2006 ▶; Su *et al.*, 2008 ▶; Zhuang *et al.*, 2008 ▶; Keizers *et al.*, 2008 ▶; Su & Otting, 2010 ▶; Hass *et al.*, 2010 ▶). In the absence of a paramagnetic metal, diamagnetic RDCs can be induced by other sources of molecular magnetic anisotropy (Zhang *et al.*, 2007 ▶) or by adding to the protein solution external orienting devices, *i.e.* large assemblies of macromolecules with strong magnetic anisotropy that can induce partial orientation in the molecule of interest by steric or electrostatic interactions (Tolman *et al.*, 2001 ▶; Chou *et al.*, 2001 ▶; Prestegard *et al.*, 2004 ▶; Chill *et al.*, 2007 ▶; Lange *et al.*, 2008 ▶; Grishaev *et al.*, 2008 ▶).

Since the 1990s, the question has been posed of whether solution NMR data can be used to refine a crystallographic structure. Crystal and solution structures can in fact differ owing to the packing forces present in crystals (Brünger, 1997 ▶; Chou *et al.*, 2001 ▶; Bertini, Kursula *et al.*, 2009 ▶; Sikic *et al.*, 2010 ▶) and/or the reduction or obliteration of solution conformational heterogeneity owing to crystallization of the protein in a single conformation (Goto *et al.*, 2001 ▶; Volkov *et al.*, 2006 ▶; Ryabov & Fushman, 2007 ▶; Tang *et al.*, 2007 ▶; Bashir *et al.*, 2010 ▶).

In 1995, PCSs were used for the first time as restraints to refine protein structures using a crystal model as the starting point (Gochin & Roder, 1995 ▶). Protocols were then presented in which the agreement of diamagnetic RDCs as well as of PCSs and self-orientation RDCs with a structural model was achieved (Chou *et al.*, 2000 ▶, 2001 ▶; Skrynnikov *et al.*, 2000 ▶; Tian *et al.*, 2001 ▶; Ulmer *et al.*, 2003 ▶; Prestegard *et al.*, 2005 ▶; Bertini, Kursula *et al.*, 2009 ▶) by restraining the backbone dihedral angles to remain close to those of the starting crystal model. Using these protocols, it was possible to verify whether the NMR data could be reproduced with minor and uniformly distributed changes in the nuclear coordinates with respect to the crystal structure, as in the case of the IgG-binding domain of protein G (Ulmer *et al.*, 2003 ▶) and of rubredoxin (Tian *et al.*, 2001 ▶), or only by allowing sizable global conformational changes, as in the case of calmodulin when free (Chou *et al.*, 2001 ▶) or bound to the calmodulin-binding peptide of the death-associated protein kinase (Bertini, Kursula *et al.*, 2009 ▶) or of the maltodextrin-binding protein loaded with β-cyclodextrin (Skrynnikov *et al.*, 2000 ▶).

PCSs and RDCs have also been used to refine the solution structures of proteins whose X-ray structures in the solid state are known by minimizing the changes in the nuclear coordinates with respect to the crystal model and at the same time matching the experimental PCS/RDC data (Gottstein *et al.*, 2012 ▶; Bertini, Ferella *et al.*, 2012 ▶). Protocols were developed for such a purpose using the *ab initio* 1 structure-calculation program *PARAMAGNETIC CYANA* (Güntert, 2004 ▶; Balayssac *et al.*, 2006 ▶).

These refinement protocols were all based on the use of structural information contained in the available crystallo­graphic model, and not on the use of the primary X-ray data. When the structures of a protein in solution and in the solid state are very similar, the NMR restraints can instead be used in conjunction with the crystallographic data, thus producing an atomic model consistent with both sets of observations.

It was often noticed that crystal models present a large number of NOE violations, while solution models obtained by NMR poorly fit the X-ray data: these discrepancies may either be owing to real differences in the molecular structure between solution and solid state, or to the different but complementary information contained in these two types of data. Joint refinements against X-ray and NOE-derived distance restraints and backbone dihedral angles are allowed by the programs *CNS* and *X-PLOR* (Brünger *et al.*, 1987 ▶) and have been performed in a number of cases. The calculations indicated that the two sets of data are largely consistent for a number of studied proteins (Shaanan *et al.*, 1992 ▶; Schiffer *et al.*, 1994 ▶; Miller *et al.*, 1996 ▶; Raves *et al.*, 2001 ▶; Tang *et al.*, 2011 ▶), mostly improving the geometry of the model in terms of the Ramachandran plot with respect to the structure calculated without NMR data. The few violating NMR restraints were mostly interpreted as real differences between the crystal and solution structures, or were ascribed to limitation of the freedom of the flexible parts of the protein occurring in the solid state and not in solution. In some cases, the joint refinement clearly provided more accurate models, for instance in the presence of regions poorly determined by X-­ray data alone owing to packing disorder within the crystal (Hoffman *et al.*, 1996 ▶) or with low- to medium-resolution diffraction data (Chao & Williamson, 2004 ▶).

PCSs and self-orientation RDCs are here proposed as additional restraints for a joint refinement together with the crystallographic data. Owing to their long-range nature, they can be more effective than NOEs as structural restraints and more helpful in disclosing structural differences between the solution and solid states. In fact, while NOEs provide local information, which is also loose in nature, the paramagnetic restraints are optimally suited to detect global structural features. The joint use of PCS/RDC restraints and X-ray data can thus indicate differences between solid-state and solution conformations or, in their absence, can be used to refine the protein structure. Both PCSs and RDCs can easily be measured in paramagnetic proteins, and possibly complemented by paramagnetic relaxation enhancements, which can also be provided for structural refinement once translated into distance restraints (Bertini *et al.*, 2008 ▶). Analogously, diamagnetic RDCs can also be used as structural restraints together with the crystallographic data.

We have here included PCSs and RDCs as structural restraints in the macromolecular crystal structure refinement program *REFMAC*5 (Murshudov *et al.*, 1997 ▶, 2011 ▶) available from *CCP*4 (Winn *et al.*, 2011 ▶). This program uses the maximum-likelihood technique to optimize the fit of atomic model parameters to X-ray crystallographic data. Agreement with the X-ray data is monitored through the *R* factor and the free *R* factor (Brünger, 1992 ▶), and agreement with the NMR data is monitored through the *Q*-factor (Cornilescu *et al.*, 1998 ▶). The use of a refinement program rather than a model-building program (Perrakis *et al.*, 1999 ▶; Cowtan, 2006 ▶; Winn *et al.*, 2011 ▶) is dictated by PCSs and RDCs being of greatest importance for structural refinement rather than in the first steps of structural calculations, when the tensor responsible for these effects cannot be safely determined from an existing protein model.

The calculations presented here on five sample cases show that the structures calculated for the catalytic domain of the protein matrix metalloproteinase 1 (MMP-1), for the protein ubiquitin and for the third IgG-binding domain of protein G (GB3) after joint refinement are in good agreement with both X-ray and NMR data; in these cases, the protein structures in the solid state and solution are apparently similar. On the other hand, in the case of calmodulin, both free and complexed with a target peptide, the joint refinement does not produce an atomic model fully consistent with both data sets, indicating the possible presence of some structural differences between the protein in solution and in the solid state.

## 2. Program implementation   

### 2.1. Paramagnetism-based restraints   

The PCS is the contribution to the nuclear chemical shift owing to the presence of a paramagnetic ion in the absence of direct electron spin-delocalization effects. It arises from the electron–nucleus through-space dipole–dipole coupling, which does not average to zero upon rotation in the presence of anisotropy in the paramagnetic susceptibility tensor. The PCS depends on the paramagnetic susceptibility anisotropy tensor χ and on the nuclear coordinates (Bertini *et al.*, 2002 ▶). For the sake of its implementation in *REFMAC*5, the equation for the PCS is written in the form
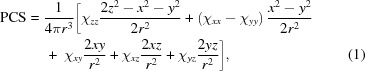
where *x*, *y*, *z* are the coordinates of the nucleus when the metal ion is at the origin and *r* is the distance between the observed nucleus and the metal ion. The axial and rhombic components of the anisotropy tensor are often defined as Δχ_ax_ = χ_*zz*_ − (χ_*xx*_ + χ_*yy*_)/2 and Δχ_rh_ = χ_*xx*_ − χ_*yy*_.

Owing to partial self-orientation of paramagnetic proteins in magnetic fields, RDCs arise that depend on the same paramagnetic susceptibility anisotropy tensor and on the orientation of the dipole–dipole coupled nuclei (Bertini *et al.*, 2002 ▶). The equation for the RDC implemented in *REFMAC*5 is 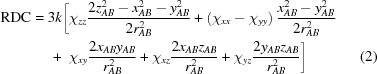
with
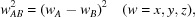


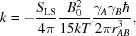
where *r*_*AB*_ is the distance between the two coupled nuclei *A* and *B*, and *S*_LS_ is the model-free order parameter introduced to take into account some average local mobility of the coupled nuclei proton vectors. RDCs do not depend on the position of the coupled nuclei with respect to the metal ion. Once a structural model of the molecule is available, and *S*_LS_ has been estimated, RDCs depend only on the five parameters defining the paramagnetic susceptibility anisotropy tensor. If these five parameters are determined from the analysis of the PCS data, RDCs are directly dependent on the orientation of the vectors connecting the coupled nuclei in a common frame. Degeneracy in the solutions can be removed by measuring several sets of RDC data arising from different paramagnetic metal ions with different principal frames of the susceptibility anisotropy tensor (Ramirez & Bax, 1998 ▶; Prestegard *et al.*, 2004 ▶; Fragai *et al.*, 2013 ▶).

Diamagnetic RDCs are described by an equation with the same form as self-orientation RDCs,
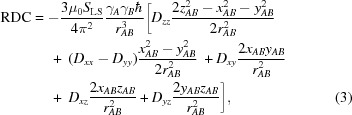
where *D_i_* are the components of the molecular-alignment tensor.

The agreement of calculated and experimental PCSs/RDCs is described by the *Q*-factors, defined as
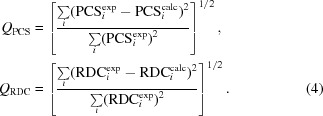



### 2.2. The paramagnetic package included in *REFMAC*5   

Protein structure refinements were performed with the modified version of the program *REFMAC*5 implementing PCS and RDC restraints. The paramagnetic package has been added starting from version 5.8, which is available from CCP4 (http://www.ccp4.ac.uk) and the Computational Crystallography Group webpage of the MRC-LMB (http://www2.mrc-lmb.cam.ac.uk/groups/murshudov).

PCSs and RDCs were calculated using (1)–(3) [Disp-formula fd2] [Disp-formula fd3]. For these calculations, a new set of H atoms was introduced. In fact, the binding distances of H atoms in X-ray libraries are different from those in NMR libraries because the hydrogen electron is not centred on the position of the nucleus but is closer to the atom to which it is attached. Therefore, the coordinates of the H atoms used for back-calculating the NMR restraints were recalculated by increasing the distance between the H atoms and their binding nuclei to the values used in the *AMBER* (Case *et al.*, 2008 ▶) library (N—^N^H distance equal to 1.02 Å; C^α^—H^α^ distance equal to 1.117 Å). This correction for the evaluation of the NMR restraints does not affect the geometric restraints in the usual X-ray refinement, which consider hydrogen positions according to the standard crystallographic library.

At each step of refinement, anisotropy/alignment tensors providing the best fits of the experimental data are estimated using a least-squares fit with the Gauss–Newton optimization technique (Nocedal & Wright, 1999 ▶). If both PCSs and RDCs arising in the presence of the same metal are present, the estimate of the tensors depends on both sets of data according to their relative weights. Thus, the magnitude and orientation of the anisotropy tensors can be driven by PCSs, if provided with a large weight, while RDCs are mostly used for the structural refinement.

The contribution of the NMR restraints (*t*) to the total optimized function is
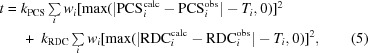
where *T_i_* is the tolerance on each of the PCS or RDC values, *w_i_* is the weight and *k*_PCS_ and *k*_RDC_ are the overall weighting factors for PCS and RDC, respectively. Table 2 shows the products of the *k*_PCS_ and *w_i_* values, indicated as ‘weight of PCSs’, and of the *k*_RDC_ and *w_i_* values, indicated as ‘weight of RDCs’, used in the calculations for the systems investigated here. In the present calculations, the tolerance was set to 0 p.p.m. for PCSs and 1 Hz for RDCs.

PCSs and RDCs must be provided in two separate files in the *PARAMAGNETIC CYANA* format. The coordinates of the paramagnetic metals related to the NMR restraints must be added to the PDB file, although these metals do not affect the X-ray contribution to the total optimized function. An instruction file is required (see Supporting Information^2^) to identify the metal and anisotropy tensors related to each set of PCSs and RDCs, to hide atoms from the X-ray data (such as the added paramagnetic metals), to provide the PCS and RDC overall weighting factors and to set further NMR options, such as joint or separated tensor estimation from PCS and RDC data. If only RDCs are used, a dummy metal must be added to the PDB file.

In order to avoid the effect of the introduction of the PCS/RDC data resulting in a worsening of the geometric parameters to fulfil these new restraints, commands have been introduced to restrain the protein structure as close as possible to the ideal geometries: two overall weighting parameters over ideal geometries of all atoms involved or not involved in the calculation of gradients and second derivatives corresponding to X-ray reflections have been added (WEIGHT REFINED_ATOMS and WEIGHT OTHER_ATOMS, respectively), and three torsion-angle restraints, pep1, pep2 and ω, have been introduced in the *REFMAC* library to restrain the planarity of the O*_i_*—C*_i_*—N_*i*+1_—C*_i_* ^α^, the C_*i*−1_—N*_i_*—C*_i_* ^α^—H*_i_* and the C^α^ *_i_*—C*_i_*—N_*i*+1_—C^α^_*i*+1_ atoms, respectively. Separate weights for these torsion-angle restraints can also be provided. In all calculations WEIGHT REFINED_ATOMS was set to 1.

## 3. Results   

Five crystal structures, the catalytic domain of MMP-1 (cMMP1), ubiquitin (Ub), the third IgG-binding domain of protein G (GB3), calmodulin (CaM) and CaM bound to the CaM-binding peptide of the death-associated protein kinase (CaM–DAPk), have been refined with *REFMAC*5 using the structure factors deposited in the PDB for entries 3shi, 3nhe, 1igd, 1exr and 1yr5, respectively. The resolution, *R* factor, free *R* factor, Ramachandran statistics and geometric parameters for the calculated structures are reported in Tables 1 ▶ and 2 ▶.

### 3.1. The protocol applied for the joint refinement from NMR and X-ray data   

As anticipated in the previous section, structure calculation with NMR data entails the strict use of ideal geometries, while structure calculation with X-ray data is more flexible. X-ray data in fact mostly depend on heavy atoms, and H atoms are typically added using library geometries^3^; on the contrary, NMR data mostly restrain the position of a few nuclei, and the coordinates of all of the remaining nuclei are determined using library geometries. In the presence of both kinds of restraints, an accurate analysis of the use of geometric restraints must be performed. Thus, to account for these factors and find the best compromise between X-ray and NMR data the weights of the geometric restraints are controlled using the new commands WEIGHT REFINED_ATOMS and WEIGHT OTHER_ATOMS (see the previous section) that control the weights of different contributions. The weights of the restraints on the three torsion angles pep1, pep2 and ω are also provided (see previous section). In all calculations performed with inclusion of the NMR data WEIGHT OTHER_ATOMS was set to 100 and the weight of ω was set to 2.

In summary, the protocol applied consists of two steps.

(i) Refinement with *REFMAC*5 performed using only X-ray data: an initial calculation is performed with an automatic setting of the weight matrix; if the calculated r.m.s.d. bond length, bond angle or chiral volume is too large to be acceptable, further calculations are performed by reducing the weight matrix value. The final values obtained for *R* factor, free *R* factor, r.m.s.d. bond length, bond angle and chiral volume are taken as reference values.
(ii) PCS and RDC data are added, the new geometric restraints are switched on and WEIGHT OTHER_ATOMS is set to 100. The weight matrix and the weights of PCS, RDC and the pep1/pep2 torsion angles are then changed in order to decrease the *Q*_RDC_ as much as possible while retaining values of the *R* factor and free *R* factor comparable to (or better than) the values calculated with X-ray data only, and r.m.s.d. bond length, bond angle and chiral volume values close to the reference values. The weight matrix is first set using the automatic *REFMAC* procedure, and then decreased in order to obtain r.m.s.d. bond length, bond angle and chiral volume values close to the reference values. The weights of the NMR data and of the pep1/pep2 angles are then increased iteratively until the free *R* factor starts increasing, and the deviations of the geometric restraints and of the pep1/pep2 angles are decreased to near to the reference values determined without the NMR data. The pep2 angle, in particular, indicates the deviation of the amide proton out of the plane defined by the other three backbone atoms, and thus monitors whether the inclusion of the ^N^H–N RDC restraints have caused deviations from ideal geometric values.

### 3.2. Catalytic domain of MMP-1 (cMMP1)   

**Figure 1:**
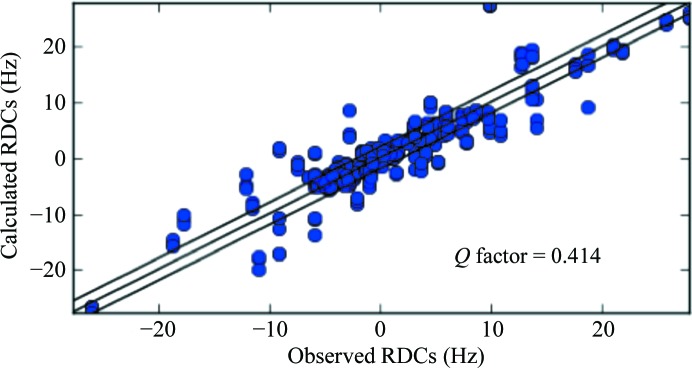
Calculated *versus* observed RDCs for cMMP1 refined with X-ray data only.

For cMMP1, PCSs of ^N^H nuclei and RDCs of the ^N^H–N pairs for three paramagnetic lanthanides (Yb^3+^, Tm^3+^ and Tb^3+^) bound to the protein through the CLaNP-5 tag were available (Bertini, Calderone *et al.*, 2012 ▶). RDCs of residues with sizable mobility, as revealed from amide-relaxation measurements (Bertini, Fragai *et al.*, 2009 ▶), were excluded; the *Q*_RDC_ of the remaining residues with respect to the crystal structure was 0.414 (see Table 1 ▶ and Fig. 1 ▶), pointing to a lack of agreement of these data with the crystal model for the protein structure in solution.

All PCSs and RDCs were then introduced as restraints in *REFMAC*5 together with X-ray data, assuming that they are all described by a unique tensor for each metal, as expected in the absence of significant motion. The presence of a small local mobility of residues was taken into account by setting a value of 0.9 for the order parameter *S*_LS_ (see equation 2). The weights of the RDC restraints were 0.3 for Yb and 0.06 for Tm and Tb, which provide larger values; the weight for the PCS restraints was 10, because they are much smaller than RDCs in absolute value. Geometric restraints on the planarity of the O*_i_*—C*_i_*—N_*i*+1_—C*_i_* ^α^ atoms (pep1) and of the C_*i*−1_—N*_i_*—C*_i_* ^α^—H*_i_* atoms (pep2) were added as described above, with the weights reported in Table 2 ▶, together with restraints on the planarity of the C^α^ *_i_*—C*_i_*—N_i+1_—C^α^_*i*+1_ atoms (dihedral angle ω = 180°), with weight set to 2.

**Figure 2:**
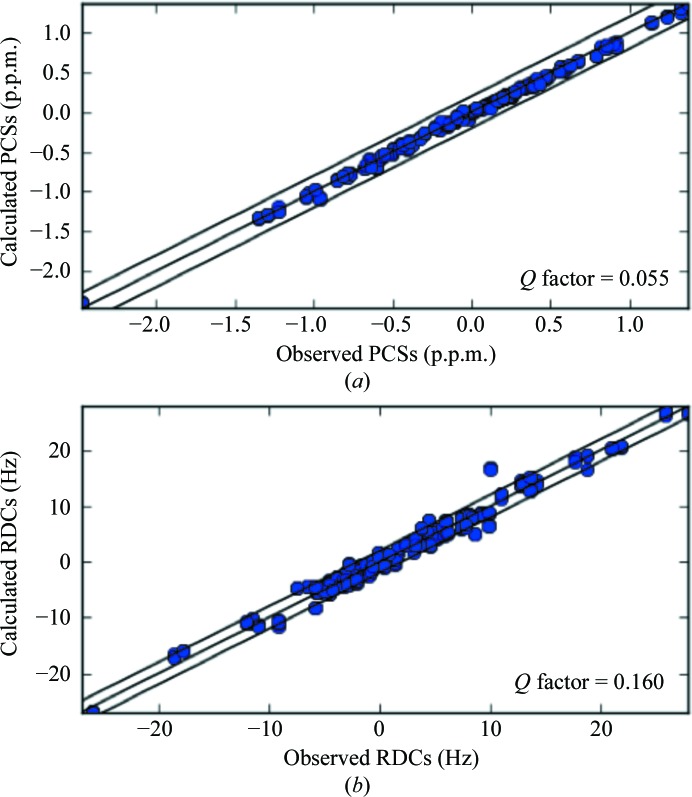
Calculated *versus* observed PCSs (*a*) and RDCs (*b*) for cMMP1 refined with both X-ray and NMR (PCSs and RDCs) data.

**Figure 3:**
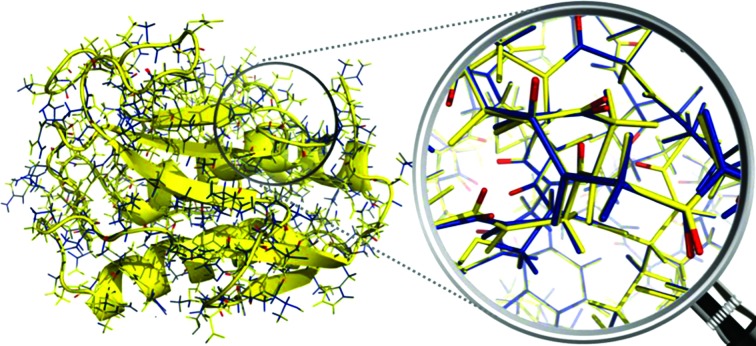
The crystal structure of MMP-1 (in yellow) and the refined structure with both X-ray and NMR data (in blue). Slight structural reorientations are visible, mainly of the bond vectors related to the observed RDCs.

The *R* factor and free *R* factor of the resulting structure are reported in Table 1 ▶, together with the *Q*_RDC_, which decreased from 0.414, calculated through a best fit of the RDC values to the structure refined with X-ray data only, to 0.160. These values indicate that the X-ray data are substantially equally well fitted in the presence and absence of the NMR data, and also point out that the structure refined with all NMR data is in good agreement with the observed RDC data, as clearly shown in Fig. 2 ▶. Fig. 3 ▶ shows that slight structural differences actually occurred, mainly in the orientation of the bond vectors related to the observed RDCs. Also, the agreement between experimental and back-calculated PCSs was satisfactory, with a *Q*_PCS_ of 0.055 (Fig. 2 ▶). The position of the metal ions was found to be in agreement with previous calculations (Bertini, Calderone *et al.*, 2012 ▶), at distances of 7–7.5 Å from the C^α^ nuclei of the tag-binding residues 132 and 136, respectively. The magnitudes of the anisotropy tensors, Δχ_ax_/Δχ_rh_, which were 8.04 × 10^−32^/−1.70 × 10^−32^, 40.9 × 10^−32^/−6.82 × 10^−32^ and −43.9 × 10^−32^/14.7 × 10^−32^ m^3^ for Yb^3+^, Tm^3+^ and Tb^3+^, respectively, were also in agreement with the previously determined values. The overall agreement of the PCSs data with a refined structure, which is only 0.039 Å (backbone r.m.s.d.) from the crystallographic structure, was indeed a clear indication that the protein conformations in the solid state and solution are similar.

In the refined structure the geometric restraints are satisfied almost equally as well as in the structure calculated before the inclusion of the PCS and RDC data. Table 2 ▶ also shows r.m.s.d. bond length, bond angle, chiral volume and pep2 violations. The increase in some of these values (and of the r.m.s.d. bond angle in particular) is in large part ascribable to the introduction of the new geometric restraints rather than to the NMR restraints. In fact, by switching on the geometric restraints with the same weights used in the all-restraints calculations, values of 0.017 Å, 2.324° and 0.116 Å^3^ for r.m.s.d. bond length, bond angle and chiral volume, respectively, were obtained without inclusion of the NMR restraints in the calculation.

The quality of the Ramachandran plot of the refined structure is also good. The number of RDC restraints with a violation larger than 2 Hz decreased from 33.8% in the structure refined with X-ray data only to 7.6% in the structure refined including the NMR restraints.

### 3.3. Ubiquitin and GB3   

In the cases of Ub and GB3, diamagnetic RDCs were available, measured using different external orienting media. For Ub, 36 sets of ^N^H–N RDCs were taken from Lange *et al.* (2008 ▶), and for GB3 five sets of ^N^H–N, C^α^–H^α^, C–C^α^ and C–N RDCs were taken from Ulmer *et al.* (2003 ▶).

**Figure 4:**
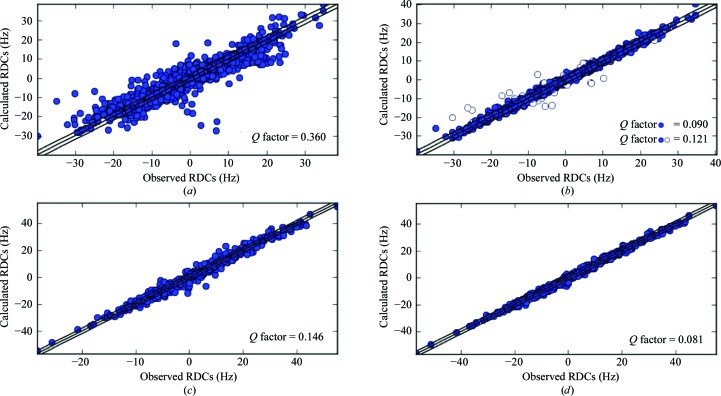
Calculated *versus* observed RDCs for Ub (*a*, *b*) and GB3 (*c*, *d*) before (*a*, *c*) and after (*b*, *d*) joint refinement against RDCs and X-ray data. The empty symbols in (*b*) show the values for residues 8 and 72.

As in the case of the catalytic domain of MMP-1, a protein structure that is in good agreement with all RDC values (see Fig. 4 ▶) can be obtained, with *R*/*R*_free_ values and geometric parameters similar to those of the structures calculated without RDCs (see Tables 1 ▶ and 2 ▶). For Ub, there are indeed very few calculated RDCs with a sizable difference from the experimental values. Interestingly, most of them are related to residues 8 and 72. Relaxation measurements (Chang & Tjandra, 2005 ▶) have indeed shown that these residues are located in regions that are likely to experience a somewhat larger mobility, although the latter is not reflected in large *B* values in the solid state (the RDCs of residues 9–10 were missing, and the RDCs of the highly flexible C-terminus, residues 73–76, were excluded from the calculations).

### 3.4. Calmodulin   

**Figure 5:**
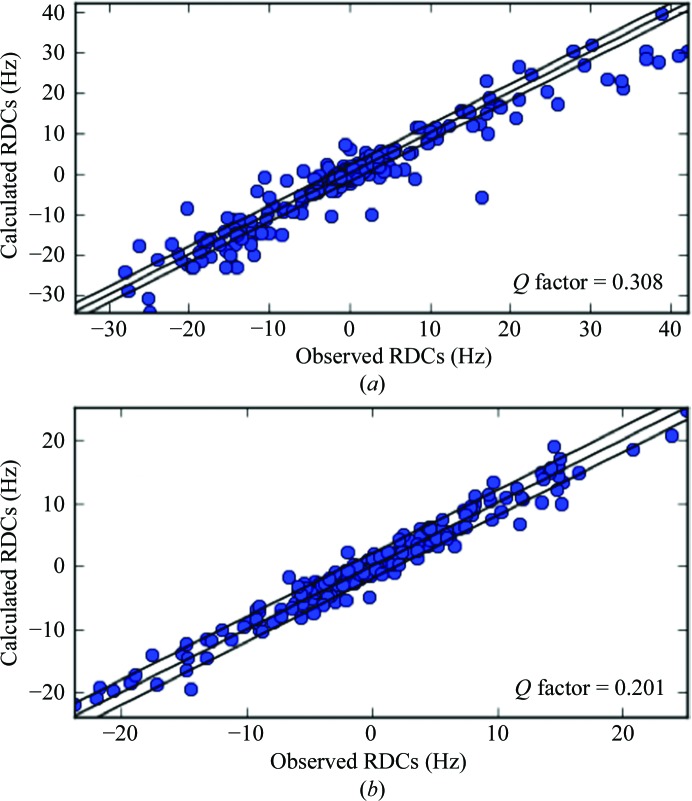
Calculated *versus* observed RDCs for CaM (*a*) and CaM–DAPk peptide (*b*) after joint refinement against NMR and X-ray data.

The structure of CaM in solution is well known to be largely different from the crystal structure because of conformational heterogeneity involving extensive reorientation of the two protein domains (Barbato *et al.*, 1992 ▶; Baber *et al.*, 2001 ▶; Dasgupta *et al.*, 2011 ▶; Bertini, Ferella *et al.*, 2012 ▶). Furthermore, even within each domain the relative positions of the four helices are somewhat different in solution with respect to the solid state, especially for the first and fourth helices of the N-­terminal domain, as shown using one set of diamagnetic ^N^H–N, C^α^–H^α^, C–C^α^, C–N and C–H^α^ RDCs (Chou *et al.*, 2001 ▶). In contrast to the cases of cMMP1, Ub and GB3, using the same set of RDCs to refine the structure together with the crystallographic restraints it was not possible in this case to achieve a good fit of the NMR data without a substantial increase in the *R*_free_ value and/or in the geometry parameters. If the weights of the RDC data and of the geometric restraints are chosen to obtain a free *R* factor and geometry parameters close to those of the structure calculated without including the RDCs, the RDCs resulted in sizable disagreement with the structure (Fig. 5 ▶ *a*). The *Q*_RDC_ in fact remained as large as 0.308. Only if substantial deviations from the crystal structure were allowed was a good fit of the RDC data (*Q*_RDC_ < 0.20) possible (Chou *et al.*, 2001 ▶).

It is also known that in the case of CaM bound to the CaM-­binding peptide of the death-associated protein kinase (DAPk) the solution and X-ray structures are different (Bertini, Ferella *et al.*, 2009 ▶). The difference is not very large, but is outside the experimental uncertainty. Three sets of PCS and ^N^H–N RDC data collected after substitution of the second calcium ion of the protein N-terminal domain alternatively with Yb^3+^, Tb^3+^ or Tm^3+^ were available. As for free CaM, a good fit of all of the RDC data was not possible without increasing the *R*_free_ value or the deviation from ideal geometric parameters. Fig. 5 ▶(*b*) shows the best fit obtained while maintaining the *R*_free_ and the geometric parameters to values similar to those obtained after refinement with X-ray data alone. The corresponding *Q*_RDC_ is 0.201. It is substantially larger than the *Q*_RDC_ of 0.14 calculated by allowing the solution structure to deviate from the crystal structure while maintaining the geometrical restraints strictly ideal. This actually suggests that the solution structure is likely to be somewhat different from the crystal structure.

## 4. Discussion   

The long-range information on the relative position of protein nuclei with respect to a common frame contained in PCSs and self-orientation RDCs, as well as in diamagnetic RDCs, has been incorporated in *REFMAC*5 to validate and refine the global fold of a protein in solution when crystallographic data are available. Self-orientation RDCs have the advantage with respect to diamagnetic RDCs of depending on the same tensor as PCSs: since PCSs are only slightly affected by local mobility and structural inaccuracies, they can provide a robust estimation of this tensor once a structural model is available, so that the RDCs can safely be used for both structural and dynamic analysis.

The fact that X-ray and NMR data can be combined to produce models that are compatible with both sets of data, in the senses that (i) the *R* factor and the free *R* factor are essentially the same as those calculated with X-ray data alone and that (ii) the NMR restraints are fulfilled with minimal violations (typically with *Q*_RDC_ < 0.2), indicates that a joint refinement against all data may provide a more reliable model for the protein in solution that is still in full agreement with the X-ray data. On the other hand, the significant restraint violations that may appear in the joint refinement indicate real differences between the protein structures in the two different states and may also provide an indication of the regions where the most significant differences occur.

In the joint minimization, it is important to select appropriate weights of the geometric restraints relative to the NMR and X-ray restraints. If large deviations from ideal geometry are allowed, full compatibility of crystal and NMR data can be achieved, but the resulting structure loses its chemical and structural integrity. Our strategy for the joint refinement is based on fixing the weights of the NMR data and of the geometric restraints (including some torsion angles) on atoms that are not refined by X-ray data to the highest possible values that still provide free *R* factors and deviations from the ideal geometric values approximately equal to those obtained in the calculations performed without including the RDCs, and at the same time providing the smallest *Q*_RDC_. This empirical procedure was successful, and an automatic search of the best values of the weights of the geometric restraints was not implemented.

The decrease of the *Q*_RDC_ to values below 0.2 after inclusion of the NMR restraints can then be used to establish when a good agreement with a single structure can be simultaneously achieved with both crystallographic and solution restraints. Among the cases considered in this study, a good agreement between the NMR restraints and the crystallographic structure was not present before the inclusion of the NMR data in the refinement protocol except in the case of GB3. Interestingly, among the cases studied, GB3 was the protein for which the X-ray structure had the highest resolution. Simultaneous refinement improved the agreement, and therefore the structure quality, even further. Upon simultaneous refinement, a satisfactory low *Q*_RDC_ was also obtained for the proteins cMMP1 and Ub when PCS/RDC data were included. Conversely, in the case of CaM the *Q*_RDC_ remained larger than 0.2, suggesting that the solution and solid-state structures of the protein differ, although in the case of CaM–DAPk peptide the situation can be considered as borderline. Although the results obtained in the above five test cases should be seen as an initial analysis that could be further optimized with a more systematic search of the weight values, the calculations performed already make us confident that we have established a reliable protocol either to safely perform a joint refinement or to assess the presence of real structural differences.

In conclusion, single refined structures that were very similar to the crystal models and were also in good agreement with the experimental NMR data could be derived for three folded compact proteins. Although some deviations from the ideal geometry of covalent bonding was allowed in the refinement, as is common when using X-ray data, it is interesting to note that even when a slight structural heterogeneity is likely to be present, all restraints are satisfied by a single protein structure. This finding is relevant to the current debate on whether disagreement between RDC data and an X-ray structure should necessarily imply the presence of sizable conformational averaging in solution (Clore & Schwieters, 2006 ▶; Lange *et al.*, 2008 ▶; Yao *et al.*, 2008 ▶). Of course, the inclusion of multiple conformations in the analysis of the data can always permit a somewhat better reproduction of the experimental RDCs. For example, in the case of Ub there are few RDC values with a deviation larger than their experimental error from the best-fit value (5.0% of the total RDCs deviate more than 2 Hz and 2.5% more than 3 Hz). RDCs, in fact, carry information on the time-averaged orientation of the corresponding vectors on time scales faster than milliseconds as well as information on their dynamic behaviour.

Distance restraints and dihedral angles for protein structure refinement are already included in *REFMAC*5. The present inclusion of long-range restraints such as PCSs and RDCs in *REFMAC*5 makes this program ideal for joint refinement against X-ray and NMR data, whatever the latter are. Since NMR is mainly sensitive to hydrogen protons and X-ray diffraction to heavy atoms, these two types of data are evidently complementary; caution should anyway be paid to the coordinates of H atoms, which must differ for the evaluation of the X-ray and NMR restraints to take care of the different distances of hydrogen nuclei and their electron cloud from the atom to which they are attached.

The availability of a joint refinement program against X-ray and NMR data could be especially valuable in the case of multidomain proteins or protein complexes where NMR data can be obtained for the individual elements. The inclusion of PCSs and RDCs together with X-ray data, besides pointing out real differences between the solid-state structure and the solution structure, can also be useful to solve structural ambiguities in cases of crystallographically poorly defined regions. Furthermore, PCSs and RDCs can indicate whether there is extensive mobility in solution which is absent in the solid state, as already pointed out in several previous papers (Tolman *et al.*, 2001 ▶; Wang *et al.*, 2007 ▶; Bertini *et al.*, 2010 ▶). The availability of protein models with the high precision typical of X-ray structures and refined using solution data may finally be useful for improving docking calculations for ligand–protein and protein–protein complexes.

## Supplementary Material

Instructions for NMR plus X-ray refinement. DOI: 10.1107/S1399004713034160/dz5299sup1.doc

Zip file with the protocols as well as coordinate files with the NMR and X-ray data together with the final refined coordinates.. DOI: 10.1107/S1399004713034160/dz5299sup2.zip

## Figures and Tables

**Table 1:** Structural parameters and quality of the refined structure for the analyzed proteins For cMMP1, three different structures are present in the crystal asymmetric unit. In this case, the values are averaged over all the structures.

	cMMP1		Ub		GB3		CaM		CaMDAPk peptide	
	X-ray (3shi)	+ PCS + RDC	X-ray (3nhe)	+ RDC	X-ray (1igd)	+ RDC	X-ray (1exr)	+ RDC	X-ray (1yr5)	+ PCS + RDC
Resolution ()	2.20		1.26		1.10		1.00		1.70	
*R* factor	0.214	0.220	0.128	0.131	0.112	0.111	0.126	0.130	0.255	0.260
*R*_free_	0.264	0.267	0.158	0.160	0.132	0.134	0.142	0.147	0.304	0.307
*Q*_RDC_	0.414	0.160	0.360	0.121	0.146	0.081	0.398	0.308	0.540	0.201
*Q*_PCS_	0.136	0.055							0.150	0.079
No. of violating RDC, PCS data†	133/393, 61/516	30/393, 4/516	678/1971	98/1971	170/877	63/877	94/314	89/314	102/214, 59/323	37/214, 23/323
Backbone r.m.s.d. ()	0.039		0.060		0.022		0.028		0.121	

† The tolerance for RDCs and PCSs was set to 2Hz and 0.1p.p.m., respectively.

**Table 2:** Structural parameters, quality of the refined structures for the analyzed proteins and weights used in the minimization procedures

	cMMP1		Ub		GB3		CaM		CaMDAPk peptide	
	X-ray (3shi)	+ PCS + RDC	X-ray (3nhe)	+ RDC	X-ray (1igd)	+ RDC	X-ray (1exr)	+ RDC	X-ray (1yr5)	+ PCS + RDC
R.m.s.d. bond length ()	0.015	0.017	0.020	0.020	0.021	0.022	0.021	0.025	0.018	0.023
R.m.s.d. bond angle ()	1.751	2.385	1.917	2.094	1.933	2.056	1.743	2.080	1.828	2.671
R.m.s.d. chiral volume (^3^)	0.129	0.112	0.128	0.116	0.227	0.098	0.091	0.113	0.112	0.155
Pep2 deviation ()	2.076	2.333	0.361	0.377	0.239	0.245	1.142	1.700	0.462	0.459
Ramachandran (%)										
Core	89.8	91.6	91.9	91.9	98.1	98.1	94.7	94.7	91.6	90.1
Allowed	9.9	7.9	7.3	7.6	1.9	1.9	5.3	5.3	7.6	8.4
Generously allowed	0.3	0.5	0.3	0.0	0.0	0.0	0.0	0.0	0.0	0.8
Disallowed	0.0	0.0	0.5	0.5	0.0	0.0	0.0	0.0	0.8	0.8
Weight matrix	0.13	0.0044	5.44	0.055	8.00	0.15	2.50	0.080	0.31	0.0073
Weight on pep1/pep2		2.5		0.5		0.2		2.0		0.6
Weight of RDCs		Yb, 0.3; Tm, 0.06; Tb, 0.06		0.1		0.1		NH, CH, CH, 0.02; CCa, CN, 0.1		Yb, 0.4; Tm, 0.08; Tb, 0.08
Weight of PCSs		10								100

## Footnotes

1 In NMR structure determination, the term ‘*ab initio*’ means that three-dimensional structures are derived directly using experimental data without a starting three-dimensional model.

2 Supporting information has been deposited in the IUCr electronic archive (Reference: DZ5299).

3 As a rule, during X-ray crystal structure refinement H atoms are added in their riding positions and they contribute to the structure-factor calculations as well as the geometry-gradient calculations. H atoms do not contribute to the gradients calculated using X-ray data.
