# Supplementary material for: Simultaneous use of solution NMR and X-ray data in REFMAC5 for joint refinement/detection of structural differences
Source: Acta Crystallogr D Biol Crystallogr. 2014 Mar 19;70(Pt 4):958–67. doi: 10.1107/S1399004713034160 (PMC4306559; doi:10.1107/S1399004713034160)
Supplement: Supplementary file 1 [file d-70-00958-sup1.doc]

*An example of REFMAC input commands:*

~/refmacgfortran

XYZIN protein.pdb

HKLIN x-ray_data.mtz

NMRIN nmr_instruction_file

PCSIN Yb_Tm_Tb.pcs

RDCIN Yb_Tm_Tb.rdc

XYZOUT refined_protein.pdb

make check NONE

make hydrogen ALL -

hout YES -

peptide NO -

cispeptide YES -

ssbridge YES -

symmetry YES -

sugar YES -

connectivity NO -

link NO

NCSR LOCAL

refi -

type REST -

resi MLKF -

meth CGMAT -

bref ISOT

ncyc 10

scal -

type SIMP -

LSSC -

ANISO -

EXPE

solvent YES

weight -

AUTO

weight refined_atoms 1.0

weight other_atoms 100.0

monitor MEDIUM -

torsion 10.0 -

distance 10.0 -

angle 10.0 -

plane 10.0 -

chiral 10.0 -

bfactor 10.0 -

bsphere 10.0 -

rbond 10.0 -

ncsr 10.0

labin FP=FP SIGFP=SIGFP -

FREE=FreeR_flag

labout FC=FC FWT=FWT PHIC=PHIC PHWT=PHWT DELFWT=DELFWT PHDELWT=PHDELWT FOM=FOM

labout FC=FC FWT=FWT PHIC=PHIC PHWT=PHWT DELFWT=DELFWT PHDELWT=PHDELWT FOM=FOM

restraint torsion include link TRANS name omega value 180 sigma 5.0 period 0

restraint torsion include link TRANS name pep1 sigma 2.0

restraint torsion include link TRANS name pep2 sigma 2.0

restraint torsion include link CIS name omega value 0 sigma 5.0 period 0

restraint torsion include link CIS name pep1 sigma 2.0

restraint torsion include link CIS name pep2 sigma 2.0

restraint torsion include link PTRANS name omega value 180 sigma 5.0 period 0

restraint torsion include link PTRANS name pep1 sigma 2.0

restraint torsion include link PTRANS name pep2 sigma 2.0

restraint torsion include link PCIS name omega value 0 sigma 5.0 period 0

restraint torsion include link PCIS name pep1 sigma 2.0

restraint torsion include link PCIS name pep2 sigma 2.0

exte dist first chain A resi 262 atom YB seco chain A resi 263 atom ZN valu 0.0 sigm 0.00001 type 1

exte dist first chain A resi 262 atom YB seco chain A resi 264 atom TB valu 0.0 sigm 0.00001 type 1

exte dist first chain A resi 264 atom TB seco chain A resi 263 atom ZN valu 0.0 sigm 0.00001 type 1

exte dist first chain G resi 262 atom YB seco chain G resi 263 atom ZN valu 0.0 sigm 0.00001 type 1

exte dist first chain G resi 262 atom YB seco chain G resi 264 atom TB valu 0.0 sigm 0.00001 type 1

exte dist first chain G resi 264 atom TB seco chain G resi 263 atom ZN valu 0.0 sigm 0.00001 type 1

exte dist first chain M resi 262 atom YB seco chain M resi 263 atom ZN valu 0.0 sigm 0.00001 type 1

exte dist first chain M resi 262 atom YB seco chain M resi 264 atom TB valu 0.0 sigm 0.00001 type 1

exte dist first chain M resi 264 atom TB seco chain M resi 263 atom ZN valu 0.0 sigm 0.00001 type 1

ANGLE 2.0

DIST 2.0

CHIRAL 2.0

TORSION 2.0

PNAME MMP1

DNAME MMP1

RSIZE 80

END

*instruction_file:*

nmr metal 1 in PCS res 262 atom YB chain A,G,M

nmr metal 2 in PCS res 263 atom ZN chain A,G,M

nmr metal 3 in PCS res 264 atom TB chain A,G,M

nmr metal 1 in RDC res 262 atom YB chain A,G,M

nmr metal 2 in RDC res 263 atom ZN chain A,G,M

nmr metal 3 in RDC res 264 atom TB chain A,G,M

nmr visible only res 262

nmr visible only res 263

nmr visible only res 264

# choose estimation between SEPAR or JOINT:

nmr estimation JOINT

nmr rtolerance 1.0

nmr weight for pcs 1.0

nmr weight for rdc 1.0
