# Supplementary material for: Simultaneous use of solution NMR and X-ray data in REFMAC5 for joint refinement/detection of structural differences
Source: Acta Crystallogr D Biol Crystallogr. 2014 Mar 19;70(Pt 4):958–67. doi: 10.1107/S1399004713034160 (PMC4306559; doi:10.1107/S1399004713034160)
Supplement: Supplementary file 2 [file d-70-00958-sup2.zip › Supporting_Information.doc]

*An example of REFMAC input commands:*

~/refmacgfortran XYZIN protein.pdb HKLIN x-ray_data.mtz NMRIN nmr_instruction_file PCSIN Yb_Tm_Tb.pcs RDCIN Yb_Tm_Tb.rdc XYZOUT refined_protein.pdb

make check NONE

make hydrogen ALL -

hout YES -

peptide NO -

cispeptide YES -

ssbridge YES -

symmetry YES -

sugar YES -

connectivity NO -

link NO

NCSR LOCAL

refi -

type REST -

resi MLKF -

meth CGMAT -

bref ISOT

ncyc 10

scal -

type SIMP -

LSSC -

ANISO -

EXPE

solvent YES

weight AUTO

!weight MATRIX 0.55

weight refined_atoms 1.0

weight other_atoms 100.0

monitor MEDIUM -

torsion 10.0 -

distance 10.0 -

angle 10.0 -

plane 10.0 -

chiral 10.0 -

bfactor 10.0 -

bsphere 10.0 -

rbond 10.0 -

ncsr 10.0

labin FP=FP SIGFP=SIGFP -

FREE=FreeR_flag

labout FC=FC FWT=FWT PHIC=PHIC PHWT=PHWT DELFWT=DELFWT PHDELWT=PHDELWT FOM=FOM

labout FC=FC FWT=FWT PHIC=PHIC PHWT=PHWT DELFWT=DELFWT PHDELWT=PHDELWT FOM=FOM

restraint torsion include link TRANS name omega value 180 sigma 5.0 period 0

restraint torsion include link TRANS name pep1 sigma 2.0

restraint torsion include link TRANS name pep2 sigma 2.0

restraint torsion include link CIS name omega value 0 sigma 5.0 period 0

restraint torsion include link CIS name pep1 sigma 2.0

restraint torsion include link CIS name pep2 sigma 2.0

restraint torsion include link PTRANS name omega value 180 sigma 5.0 period 0

restraint torsion include link PTRANS name pep1 sigma 2.0

restraint torsion include link PTRANS name pep2 sigma 2.0

restraint torsion include link PCIS name omega value 0 sigma 5.0 period 0

restraint torsion include link PCIS name pep1 sigma 2.0

restraint torsion include link PCIS name pep2 sigma 2.0

PNAME MMP1

DNAME MMP1

RSIZE 80

END

*instruction_file:*

nmr metal 1 in PCS res 262 atom YB chain A,G,M

nmr metal 2 in PCS res 263 atom ZN chain A,G,M

nmr metal 3 in PCS res 264 atom TB chain A,G,M

nmr metal 1 in RDC res 262 atom YB chain A,G,M

nmr metal 2 in RDC res 263 atom ZN chain A,G,M

nmr metal 3 in RDC res 264 atom TB chain A,G,M

nmr visible only res 262

nmr visible only res 263

nmr visible only res 264

\# choose estimation between SEPAR or JOINT:

nmr estimation JOINT

nmr rtolerance 1.0

nmr weight for pcs 1.0

nmr weight for rdc 1.0

*To activate the* pep1 *and* pep2 *restraints, the following lines must be added in the CCP4 library file* mon_lib_list.cif*, located in the folder* ccp4-#.#.#/lib/data/monomers/list.

*Inside the file, you need to locate the* data_link_TRANS *part and add the definition of the two torsion angles* pep1 *and* pep2*. Therefore, the following part:*

loop_

_chem_link_tor.link_id

_chem_link_tor.id

_chem_link_tor.atom_1_comp_id

_chem_link_tor.atom_id_1

_chem_link_tor.atom_2_comp_id

_chem_link_tor.atom_id_2

_chem_link_tor.atom_3_comp_id

_chem_link_tor.atom_id_3

_chem_link_tor.atom_4_comp_id

_chem_link_tor.atom_id_4

_chem_link_tor.value_angle

_chem_link_tor.value_angle_esd

_chem_link_tor.period

TRANS	psi	1 N	1 CA	1 C	2 N	160.00 30.0 2

TRANS	omega	1 CA	1 C	2 N	2 CA	180.00	5.0 0

TRANS	phi	1 C	2 N	2 CA	2 C	60.00 20.0 3

*must be replaced with the following part (with the last two lines added):*

loop_

_chem_link_tor.link_id

_chem_link_tor.id

_chem_link_tor.atom_1_comp_id

_chem_link_tor.atom_id_1

_chem_link_tor.atom_2_comp_id

_chem_link_tor.atom_id_2

_chem_link_tor.atom_3_comp_id

_chem_link_tor.atom_id_3

_chem_link_tor.atom_4_comp_id

_chem_link_tor.atom_id_4

_chem_link_tor.value_angle

_chem_link_tor.value_angle_esd

_chem_link_tor.period

TRANS	psi	1 N	1 CA	1 C	2 N	160.00 30.0 2

TRANS	omega	1 CA	1 C	2 N	2 CA	180.00	5.0 0

TRANS	phi	1 C	2 N	2 CA	2 C	60.00 20.0 3

TRANS	pep1	1 CA	1 C	1 O	2 N	0.00	1.0 2

TRANS	pep2	1 C	2 N	2 H	2 CA	0.00	1.0 2

*Also* PTRANS*,* CIS *and* PCIS *must be defined by adding the following lines in the corresponding parts:*

PTRANS	pep1	1 CA	1 C	1 O	2 N	0.00	1.0 2

PTRANS	pep2	1 C	2 N	2 H	2 CA	0.00	1.0 2

CIS	pep1	1 CA	1 C	1 O	2 N	0.00	1.0 2

CIS	pep2	1 C	2 N	2 H	2 CA	0.00	1.0 2

PCIS	pep1	1 CA	1 C	1 O	2 N	0.00	1.0 2

PCIS	pep2	1 C	2 N	2 H	2 CA	0.00	1.0 2
